# Can we detect fibrofatty band in patients with bowel obstruction on CT scan?

**DOI:** 10.1259/bjrcr.20210016

**Published:** 2021-07-08

**Authors:** Chunhei Li, Robert Pallas, Natasha Frewer, Julie Cornish, Rwth Ellis-Owen

**Affiliations:** 1 Cardiff University School of Medicine, Wales, United Kingdom; 2 Department of Radiology, Cardiff and Vale University Health Board, Wales, United Kingdom; 3 Department of Pathology, Cardiff and Vale University Health Board, Wales, United Kingdom; 4 Department of Colorectal Surgery, Cardiff and Vale University Health Board, Wales, United Kingdom

## Abstract

Fibrofatty bands are composed of adipose tissue and connective tissue and can tangle around the bowel and caused intestinal obstruction. Currently, there is a lack of radiological teaching or guidance on how to identify fibrofatty band in patients with bowel obstruction. The true incidence of fibrofatty band-induced bowel obstruction is likely to have been overlooked. We present a case series of patients with fibrofatty bands with different features and aim to highlight the key radiological findings that may help in the radiological diagnosis. We advocate that these features should be incorporated into the current algorithm for radiologist when assessing scan images of patients with intestinal obstruction.

## Introduction

Small bowel obstruction (SBO) accounts for around 16% of all annual surgical admission^1^ in the western world. 75% of all cases of SBO are caused by abdominal adhesions, followed by volvulus and hernia.

CT scan is the preferred modality to aid the diagnosis and assess the severity of obstruction.^2^ In the absence of high-degree SBO (features includes intestinal ischemia, necrosis, perforation, sepsis), the management tends to be solely conservative, which includes nasogastric tube insertion and Intravenous fluid replacement.^3–5^ However, one-third of the patients would fail to respond to conservative management and eventually require surgery to relief the intestinal obstruction.^5^ The decision and timing for surgery is ultimately determined by surgeon’s clinical judgement in combination with radiological findings. There has been a rise in the number of CT scans performed on patients with small bowel obstruction, partly as a result of the recent National Audit of Small Bowel Obstruction study.^5^ In our institution, we had noticed that there was an increase in cases of extraluminal band identification for patients who had CT proven SBO. These extraluminal bands are rarely reported in the existing literature and are recognised as fibrofatty band following surgery.^6^ From our experience, the cohort of patients with fibrofatty bands did not respond to the conservative management alone, and ultimately required surgery. Early detection of these bands may allow better informed clinical decision for surgeons and prompt surgical intervention to avoid waiting from conservative management.^5^ Fibrofatty bands are more likely to be amenable to a laparoscopic approach and avoid the need for a midline incision, allowing for quicker recovery.

Upon review of these cases with the radiology team, there are recurring and distinctive radiological features that could help to identify these fibrofatty bands on the CT scan. In this case series, we highlight these features and would recommend them to be integrated into a radiological algorithm upon reviewing the scan for SBO.

## Case series

### Case 1

A 52-year-old male presented with 1-day history of sudden onset right iliac fossa pain. No nausea and vomiting were reported. Three weeks prior to this admission, he had an emergency right hemicolectomy for a perforation related to stricture at terminal ileal disease caused by Crohn’s disease. On examination, his abdomen was tender without gross distension. The only abnormality in his blood results was the elevated white blood cell count. An intravenous contrast-enhanced CT was performed and demonstrated a moderate length segment of oedematous and thickened wall at neoterminal ileum, which was interpreted as recurrent neoterminal Crohn’s ileitis.

**Figure 1.**
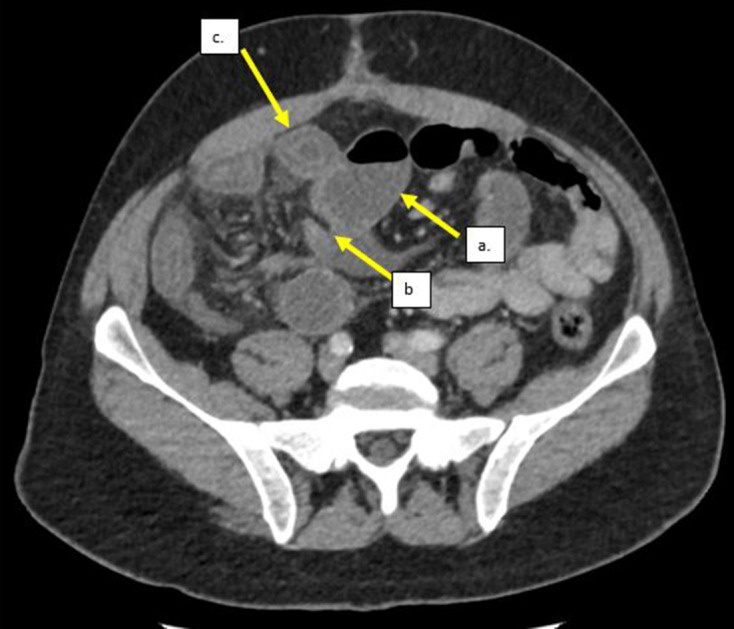
An axial enhanced CT showing: a. Dilated small bowel up stream to the transition point. b. Acute transition point caused by a fat density band indenting the bowel. c. Non-dilated but edematous small bowel downstream to the transition point. This was initially misinterpreted as recurrent Crohns but was re-reported as congestion and edema downstream to the fibrofatty band obstruction.

The patient was then treated with i.v. fluids and analgesia, but the pain was intractable despite the treatment. On second review of the CT scan by the radiologist, it was noted that there was an acute transition from dilated to non-dilated small bowel with a linear fat density band running through the mesentery causing an indentation on the small bowel at the transition point (Figure 1a), these findings were in keeping with acute small bowel obstruction secondary to an adhesive fibrofatty band. The edematous small bowel was reinterpreted as being related to congestion downstream to the obstruction. A decision was made to proceed to theater to explore the abdomen, where an adhesive fibrofatty band was identified at the mid-ileum and bowel appeared to be ischemia with edematous in the theater. The time from admission to surgery was less than a day. A band division was performed and the bowel condition improved and was viable. No histology was sent. His pain resolved quicklyand he was discharged with no symptoms to suggest active small bowel Crohn’s disease.

### Case 2

**Figure 2.**
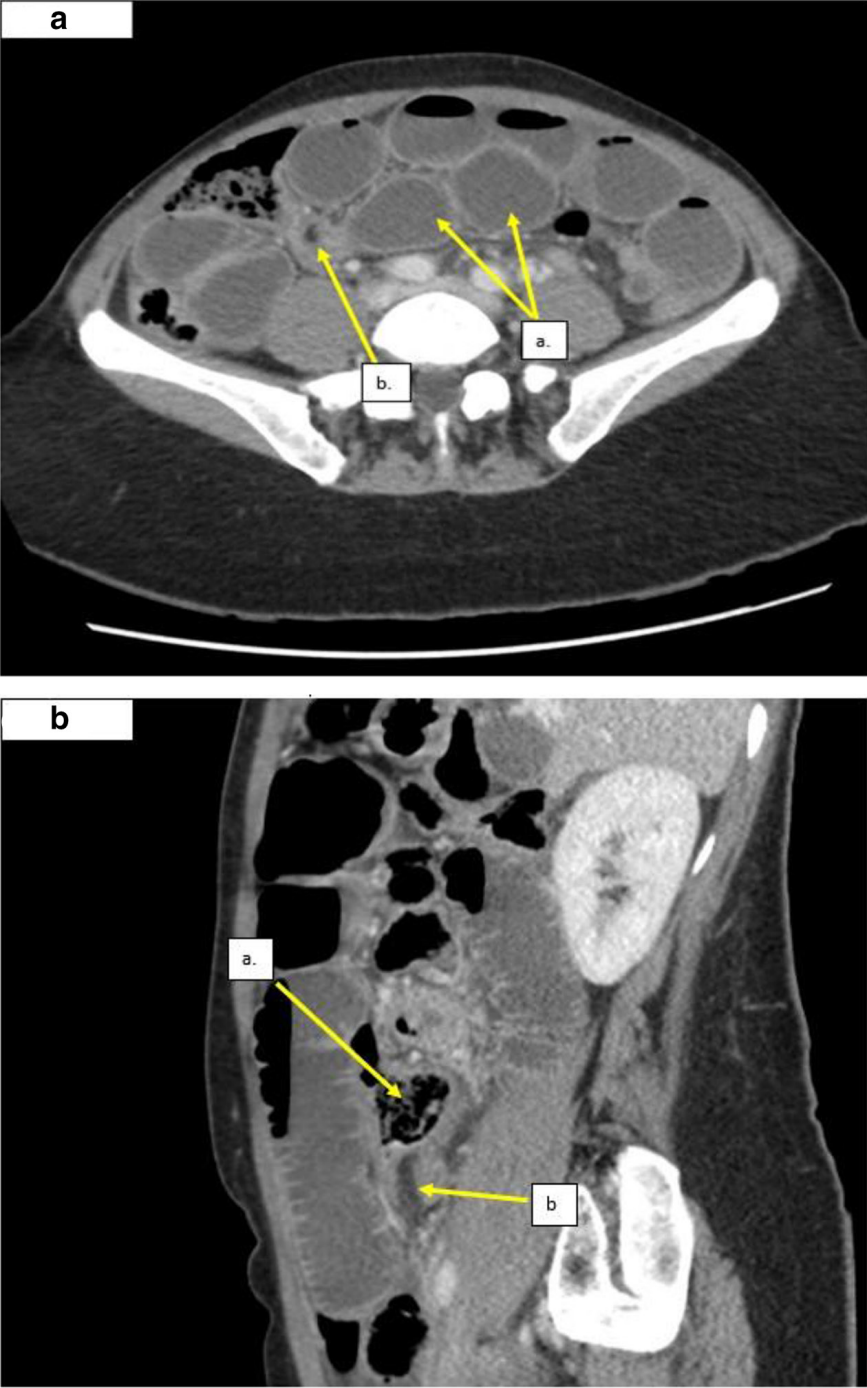
a. Axial CT images showing multiple loops of dilated and fluid-filled small bowel with a linear fat density structure at the transition point (Case 2) a. Dilated, fluid filled small bowel loops. b. Cross-section through the fibrofatty band demonstrating a fat density center. b. Sagittal images from the same CT demonstrating: a. Small bowel feces within the obstructed small bowel up stream of the acute transition point. (**b.**) Linear fat density band at the acute transition point, the distal small bowel was collapsed downstream to this.

A 49-year-old female presented with 3-day history of lower abdominal pain. She had an absolute constipation for 6 days. Her only previous surgical history was 3 Cesarean sections. Examinations were unremarkable except lower abdominal tenderness. Blood investigations were normal. She was initially referred for an ultrasound scan with no other treatment, however she then developed bilious vomiting and worsening of her abdominal symptoms. A CT abdomen/pelvis demonstrated the presence of dilated, fluid-filled small bowel loops leading to a transition point in the right iliac fossa. A linear fat density structure was seen anteriorly across over the collapsed bowel at the transition point which was in keeping with a fibrofatty band as the cause of the small bowel obstruction (Figure 2). In this case, the fibrofatty band had more of a fibrous component allowing the band to be visualised better, with the fat density center allowing confident diagnosis of a fibrofatty band on CT. The patient immediately underwent urgent surgery. The time from admission to surgery was 4 days. An adhesion fibrofatty band was discovered in the paracolic area as suggested by CT scan. Band division was performed. No histology was sent. The patient completely recovered within 72 h of surgery and was discharged.

### Case 3

**Figure 3.**
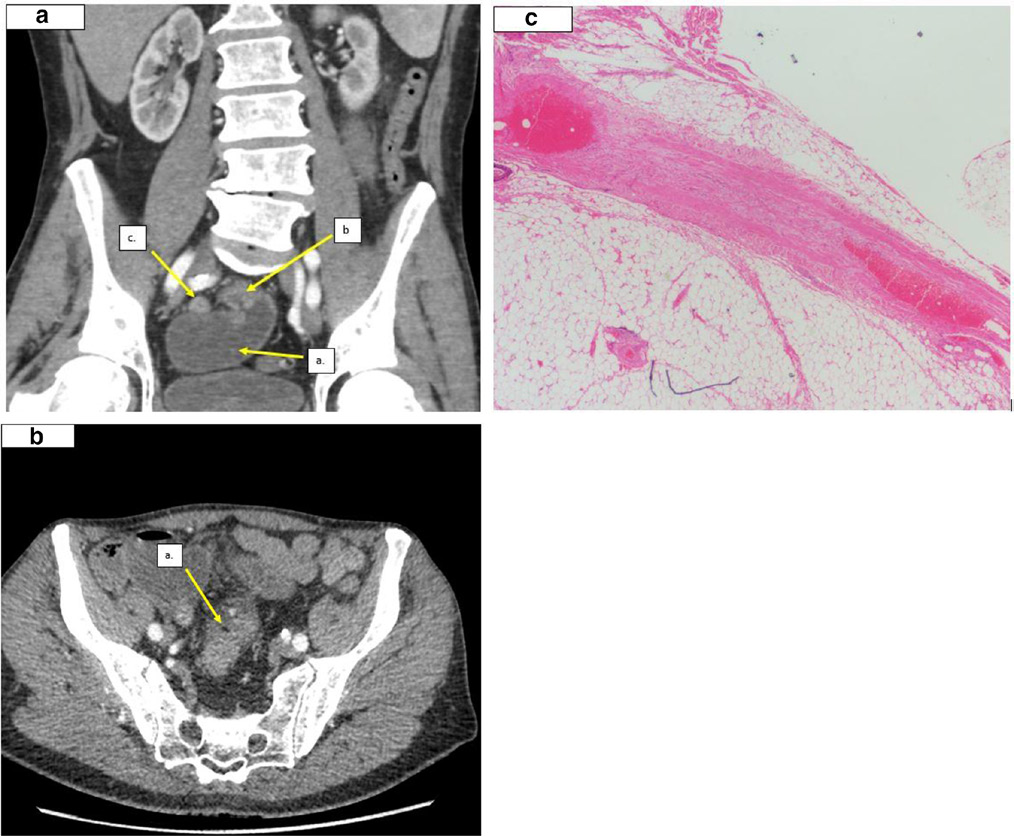
a. coronal CT scan demonstrating: (a) Dilated fluid filled small bowel loops. b. Acute transition point in the pelvis, a fibrofatty band was not well appreciated on the coronal images. c. Collapsed small bowel loops downstream to the transition point. b. Axial CT images demonstrating: (a). Fat-density band indenting the small bowel at the acute transition point. c. Photomicrograph showing a fibrofatty band in a patient with a previous history of surgery. Specimen is composed of fibrous connective tissue and fat. Sections were H&E stained (magnification x10).

A 76-year-old male presented with several days’ history of progressive worsening acute abdominal pain. He had experienced a few similar episodes previously, but the symptoms had settled spontaneously without any medical intervention. His past surgical history included appendicectomy. Blood tests showed a deranged renal function with mildly elevated inflammatory markers in keeping with acute kidney injury. His lactate level was normal. A CT abdomen/pelvis reported acute small bowel obstruction with small bowel feces proximal to an acute transition point in the distal ileum. A linear fat density band could be seen to traverse the bowel at the transition point suggestive of a fibrofatty band. Surgery was performed and a fibrous band was found and divided. The time from admission to surgery was less than a day. Histology confirmed that the adhesion was composed of fibrofatty tissue (Figure 3c). The patient recovered well and was fully discharged after 72 h.

### Case 4

An 87-year-old male presented with 3 days history of sharp, radiating upper abdominal pain. He described constipation, passing only two episodes of small volume hard pellets and vomiting about 1 liter of brown fluid on that day. His past medical history included epilepsy, a cholecystectomy, and a right inguinal hernia repair. He was hemodynamically stable and found to have a mildly distended abdomen with upper abdominal tenderness. A CT scan was performed which reported a fibrofatty band and small bowel obstruction. He underwent surgery and band division. The time from admission to surgery was less than a day. He had a complete resolution of symptoms and was discharged 2 days after his operation.

**Figure 4.**
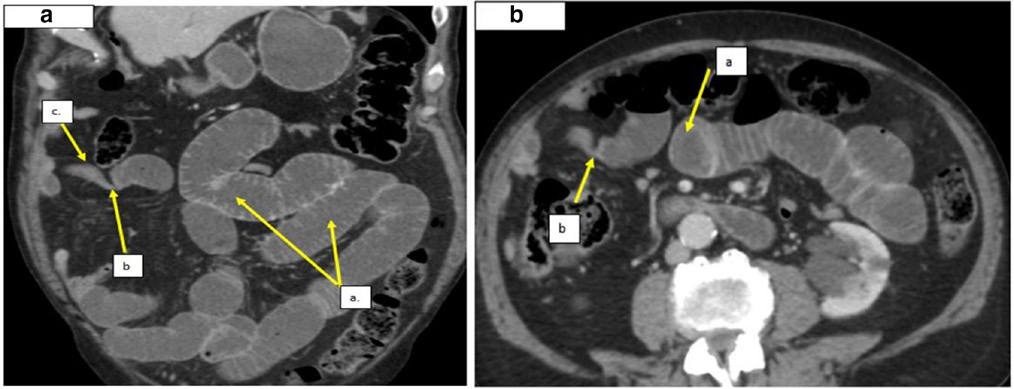
a. Axial CT images and Figure 4b, Coronal CT images, demonstrating: (a) dilated fluid filled small bowel loops upstream to the acute transition point. a. Fat density band indenting the bowel lumen at the acute transition point. b. Collapsed small bowel distal to the transition point.

### Case 5

**Figure 5.**
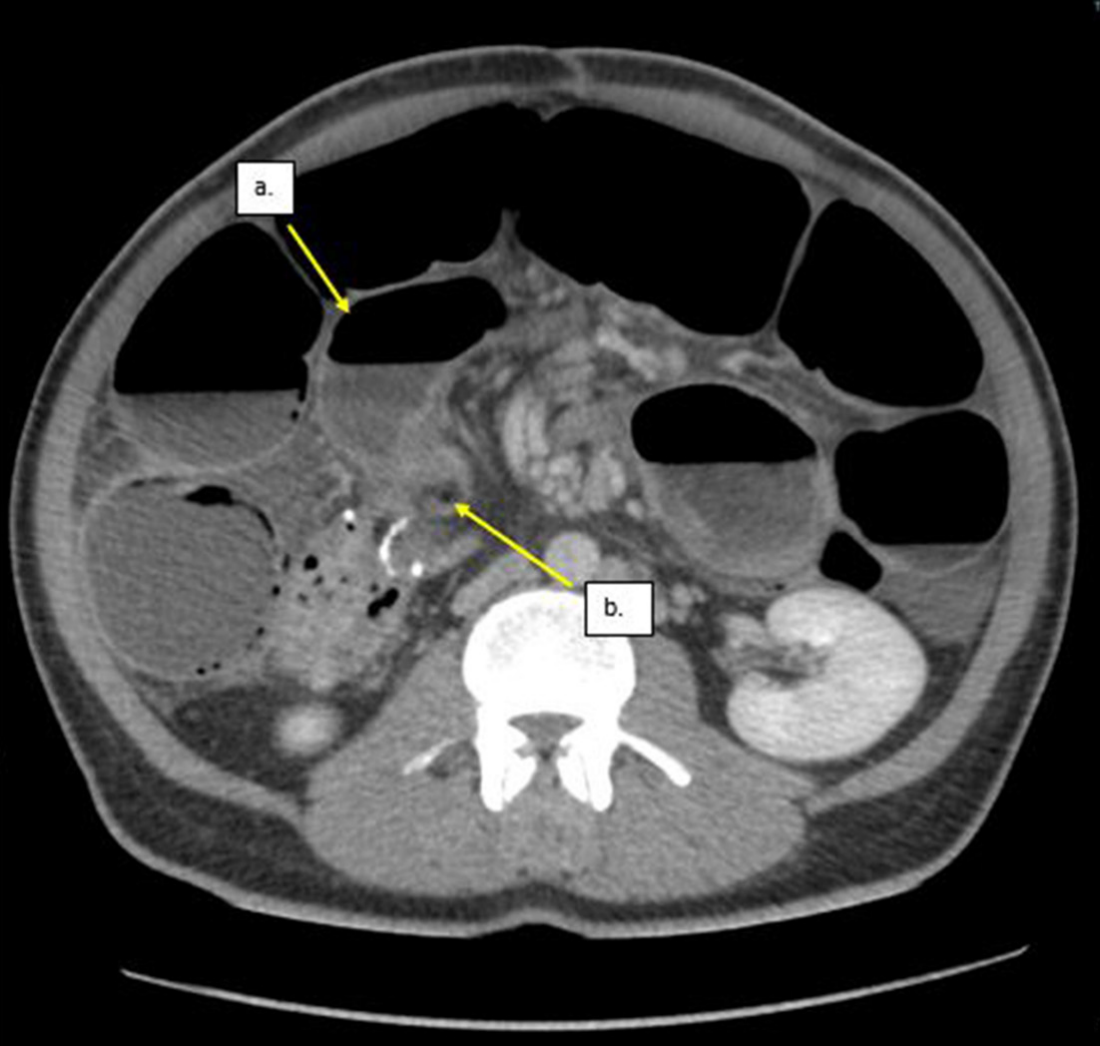
Axial CT demonstrating: (a) dilated fluid filled small bowel loops up stream to the acute transition point. b. Twisting of the small bowel around a fat density structure at the transition point, this fat density structure was demonstrated to represent a linear band on coronal images.

A 35-year-old male was presented with 1 day history of vomiting and increasing abdomen pain. He had laparotomy 3 months ago for a strangulated bowel ischemia that was caused by Meckel’s diverticulum. CT on this admission demonstrated acute small bowel obstruction with a long linear band consisting of linear soft tissue with central fat density traversing the bowel at the transition point, immediately proximal to the small bowel suture line from previous surgery (Figure 5) (Supplementary Material 1). He was initially managed conservativity with no response and proceeded to surgery. During the operation, a thick band was found between the omentum and small intestinal mesentery. The small bowels were grossly dilated intraoperatively but fully viable. Division of band was performed. The time from admission to surgery was 2 days. No histology was sent but he was discharged on Day 5.

Supplementary Material 1.

### Case 6

**Figure 6.**
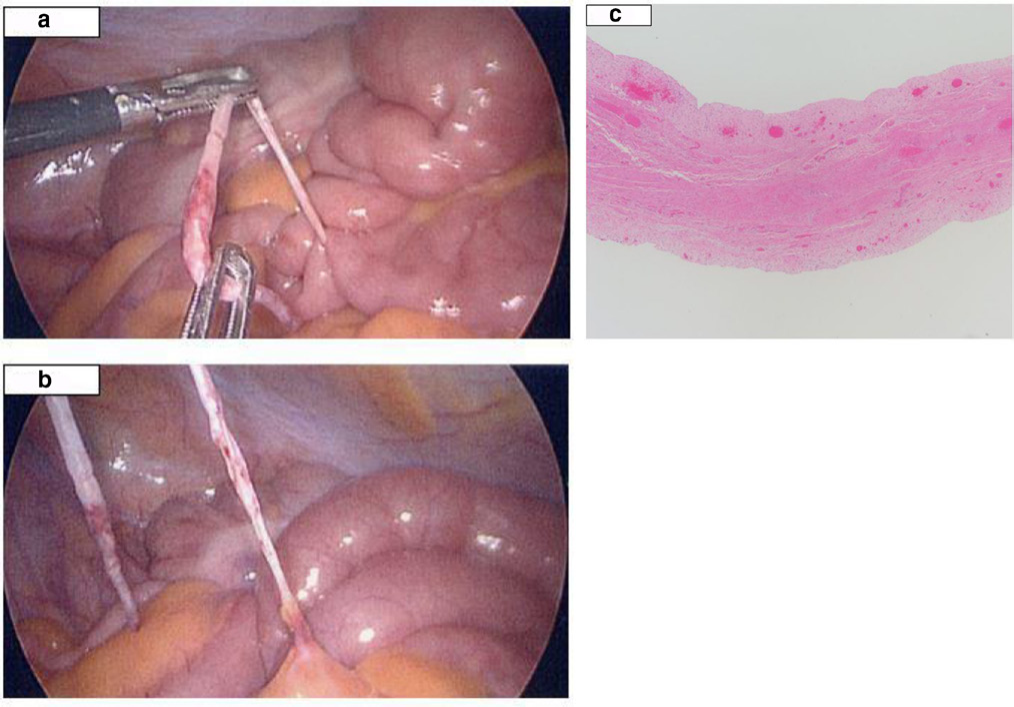
(a,b) Fibrofatty band images were taken during intraoperatively. The fibrofatty band is demonstrated. c Photomicrograph showing a fibrofatty band in a patient with no previous history of surgery. Specimen is composed of fibrous connective tissue. Sections were H&E stained (magnification x10).

A 27-year-old male who presented with a 1 day history of severe right iliac fossa pain (RIF), no associated vomiting or nausea were reported. The patient continued to complain of discomfort, worse on movement. He did not have any past surgical or medical history. On examination, Rovsig sign was positive with RIF tenderness. Urine dip was unremarkable. The blood results demonstrated a mildly raised CRP 82. This was felt to be in keeping with appendicitis and the patient had a diagnostic laparoscopy without CT scan being performed. The time from admission to surgery was less than a day. At surgery, the appendix appeared macroscopically normal. There was a fibrofatty band adhesion of small bowel mesentery to the abdominal wall causing small bowel obstruction at mid-jejunum (Figure 6a–b). Surgical division of band was performed, and the patient went home after 24 h.

The histology result reported the presence of cellular fibrous connective tissue, collagen, smooth muscle fibers and adipose tissue (Figure 6c).

## Discussion

In this case series of six patients with fibrofatty band (age ranging from 27–87 years old), all presented with abdominal pain but not all symptoms were typical of small bowel obstruction. Of those, five patients had a history of abdomen surgery, whereas one has no surgical history. Fibrofatty bands are highly elastic and stretchable, so patients with band-induced obstruction are unlikely to settle with conservative management alone. The average time from admission to surgery in this case series was 1.6 days, which was less than the national average time of 3.9 days in the UK ^5^ Identification of fibrofatty bands by radiologist could help to prompt early surgical intervention.

The occurrence of peritoneal adhesions is well documented in the literature in patients after abdomen surgery,^7^ with fibrofatty bands being recognied as a type of adhesion which are composed of adipose tissue and connective tissue. When these fibrofatty bands are found in the patients without prior operation, also known as “virgin”, abdomen, they are referred to ‘congenital fibrofatty band’. The exact pathogenesis of adhesion remains inconclusive. Fibrin deposition occurred as part of the normal healing process, however insufficient fibrinolysis at later stage could results persistent and excessive fibrin, which can later transform into fibrocollagenous tissues and form adhesion. The acquirement of fibrofatty bands could be hypothesised as further fibrogenesis process from those residual fibrocollagenous tissues.^7^ It remains unclear why some individuals are affected by the formation of bands.

In contrast to the acquired fibrofatty band, little is known for the congenital fibrofatty band. On reviewing literature, the origin of fibrofatty band is thought to be related to the vitelline duct.^6,8–10^ The vitelline duct is part of the omphalomesenteric duct that connects the yolk sac to the primitive mid-gut during the embryonic development, which received its nutrition from the vitelline artery. Vitelline duct usually disappears by 7th −9th week of gestation and failure of its removal, which occurs in 2% of the population, can lead to a variety of congenital anomalies (such as Meckel diverticulum, patent vitelline duct and fibrous fatty band). These congenital remnants are established causes of small bowel obstruction in infancy. However, most patients with fibrofatty band would remain asymptomatic throughout their lives.^8^ Incidental radiological detection hence is possible.

The intestinal obstruction occurred when the fibrofatty band wrapped around the intraperitoneal small intestinal cumulating in its symptom’s presentation. The distal ileum seems to be a common site for SBO.^3,4^ There were no apparent precipitating factors triggering the occurrence of SBO in our patients. One previously reported case at our institution was suspected to be precipitated by running a marathon, in this case it is postulated that the rigorous exercise and increase mesenteric vascular flows resulted in entanglement.^6^

Recent evidence from the National Small Bowel Obstruction audit advises prompt CT for patients presenting with SBO and allows greater opportunity for identification of fibrofatty bands.^5^ These are potentially amenable to laparoscopic division, compared to more dense abdominal adhesions from previous surgery, and can guide surgeons’ approach to the patient. Radiological input is important to help identifying the presence of the fibrofatty band as this could influence the decision-making for surgery, allowing better pre-operative planning. Prompt identification potentially aids laparoscopic division of the band with reduced small bowel dilatation. The fibrofatty band has a defined band structure that is potentially easy to identify and divide laparoscopically. Unlike volvulus and hernia, confidently identifying a fibrofatty band on a CT is challenging and may be overlooked by the radiologist, as the density of the tissues within the band is similar to those of the surrounding mesentery and omentum as demonstrated by this cases series.

Careful interrogation of any acute transition point in the context of SBO on all available CT reformats can help identify the pathognomonic linear fat density within a fibrofatty band that will allow confident diagnosis of fibrofatty band on CT. This can help to differentiate fibrofatty band obstruction from other types of adhesive obstruction. Radiological features that can help identify fibrofatty bands on CT include identifying acute transition point in the bowel with central area of fat density at the transition, occasionally a more focal band of well-defined fat surrounded by some soft tissue can be seen to coursing between two abdominopelvic structures, running through the transition point in the bowel and indenting or kinking the bowel.

To conclude, recognizing these fibrofatty bands in patients with bowel obstruction is essential to prompting early surgical intervention. There is no focus in the current radiological curriculum to identify the fibrofatty band, which remains as a diagnosis of exclusion. Therefore, we challenge the radiologist to actively look for the fibrofatty band upon reviewing the CT scan and we have highlighted few radiological features l to help detecting thefibrofatty band.

## Learning points

- Radiologist should actively look for fibrofatty band in the small bowel obstruction using all available CT reformat.
- The tissues density of the fibrofatty band is similar to the surrounding mesentery and omentum.
- Compression from fibrofatty band can cause edema in the downstream intestinal loop which can mimic the appearance of Crohn’s disease.
- The presence of fibrofatty band should prompt surgical intervention.
